# Venous Thromboembolism in Hospitalized Patients With Surgical Breast Cancer: Risks and Outcomes

**DOI:** 10.7759/cureus.42096

**Published:** 2023-07-18

**Authors:** Omobolaji Ayandipo, Oluwasanmi Ajagbe, Adefemi Afolabi, Temidayo Ogundiran, Akin Orunmuyi, Olufunmilayo Soneye

**Affiliations:** 1 Oncology, University of Ibadan, Ibadan, NGA; 2 Surgery/Oncology, University College Hospital, Ibadan, NGA; 3 Surgery/Endocrine, University College Hospital, Ibadan, NGA; 4 General Surgery/Oncological Surgery, University College Hospital, Ibadan, NGA; 5 Nuclear Medicine, University College Hospital, Ibadan, NGA; 6 Surgery, Federal Medical Center, Lagos, NGA

**Keywords:** risk, nigeria, breast cancer, caprini tool, venous thromboembolism

## Abstract

Background

The Caprini risk assessment model has been validated in breast cancer surgery patients. However, its utility in our population has not been described. This study evaluated the benefits and risks of the Caprini risk stratification tool and the incidence of venous thromboembolism (VTE) in the 30-day postoperative period among surgical female patients with breast cancer who were hospitalized during their treatment.

Methodology

This is a retrospective review of prospectively collected data of all surgical patients with histologically confirmed breast cancer who were hospitalized between January and December 2018. Caprini score, treatment information, and 30-day outcome of prophylaxis were collated and analyzed using SPSS version 26 (IBM Corp., Armonk, NY, USA).

Results

A total of 167 female patients with breast cancer aged 19 to 75 years were hospitalized during the study period. All patients had invasive ductal carcinoma, and the majority (76.6%) were premenopausal. Two fatal VTE events occurred during hospitalization, giving a 30-day incidence of 1.2%. There was no adverse event from chemoprophylaxis.

Conclusions

VTE is rare in hospitalized surgical patients with breast cancer undergoing routine pharmacologic and mechanical prophylaxis. The Caprini tool can identify extremely low-risk patients who require no prophylaxis.

## Introduction

Venous thromboembolism (VTE) is a common and potentially fatal event in the surgical management of cancer [1]. Cancer patients undergoing surgery have twice the risk of postoperative nonfatal deep venous thrombosis (DVT) and three times the risk of fatal pulmonary embolism compared with noncancer patients undergoing similar surgery [2]. Previous studies have identified the cancer-related, patient-related, and treatment-related factors that contribute to the development of VTE in cancer patients [3]. This understanding has facilitated the development of VTE prediction models and thromboprophylaxis in the surgical management of cancer. Typically, these initiatives are aimed at documenting the prevalence of VTE and justifying the use of thromboprophylaxis in the hospital setting.

Female breast cancer is the leading cause of cancer morbidity and mortality in women worldwide. Although breast cancer is associated with an increased risk of VTE, recent studies report a lower risk of VTE after breast surgery compared to major operations of the abdomen and pelvis performed for cancer, particularly in patients who are treated on clinical pathways with mechanical anti-embolism devices and early postoperative ambulation [4]. Nevertheless, the risk of VTE in breast cancer has been reported to be highest in the first month following surgery [5]. Several factors contribute to the development of VTE after surgery in breast cancer. Patient-related factors include age >65, race, and the presence of comorbidities [4]. The type of surgical procedure, operative time under general anesthesia >3 hours, increased length of hospital stay, recent surgery within 30 days before the breast operation, and cancer therapy (hormonal therapy) constitute some of the treatment-related risk factors for VTE in breast cancer. Similarly, tumor-related characteristics (histological subtype and advanced stage of disease) play a role in the development of VTE in breast cancer [4].

Studies have suggested that VTE is a rare event in patients who are treated on clinical pathways [6]. However, this has not been established in sub-Saharan Africa.

This study investigated the benefits and risks of the Caprini risk stratification tool and our institutional VTE prevention program for hospitalized surgical breast cancer patients, including chemoprophylaxis during hospitalization with low-molecular-weight heparin (LMWH; Enoxaparin), for all patients and follow-up at 30 days post discharge from hospital.

## Materials and methods

This is a retrospective review of collated data in a cohort of Nigerian women with histologically confirmed breast cancer who required surgery or other interventions irrespective of stage who were admitted to the surgical oncology ward of the University College Hospital (UCH), Ibadan, from January to December 2018, were included in the study. Patients with breast cancer who did not indicate in-hospital care were excluded from the study. UCH Ibadan is a major oncology referral hospital in Southwest Nigeria, and the surgical unit is a major surgical oncology training center in Nigeria.

Inclusion criteria

Inclusion criteria for this study consist of all patients with a histologically confirmed diagnosis of breast cancer, as well as breast cancer patients requiring hospital admission during the study period.

Exclusion criteria

Exclusion criteria include breast cancer patients who do not have a medical indication for hospitalization.

The surgical oncology unit implements a routine VTE protocol for hospitalized patients comprising a Caprini risk stratification tool, clinical care pathway (a short pre- and postoperative in-patient stay and clotting profile assay) chemoprophylaxis during hospitalization with LMWH (Enoxaparin), and mechanical prophylaxis (thigh-length thromboembolic deterrent, or TED, compression stockinet use for all patients, and early commencement of limb physiotherapy), and follow-up at 30 days after discharge from hospital. Patients diagnosed with VTE received multidisciplinary management with hematologists, pulmonologists, radiologists, and other specialists, as required.

Primary recorded data from clinical case notes and ward admission register, including demographics, histology and stage of cancer, type of surgical procedure, and outcome of VTE prophylaxis, were collated. Participants were further classified based on menopausal status at the time of enrolment (premenopausal or postmenopausal) and disease stage according to the American Joint Committee on Cancer (AJCC) system for breast cancer, while results of the Caprini questionnaire were classified according to the risk categories. The outcome of interest was the occurrence of a VTE event or adverse effect of VTE prophylaxis (SC Enoxoparin 40 mg daily; hematoma and prolonged bleeding) within 30 days of hospitalization. Data were analyzed using SPSS version 26 (IBM Corp.). The results are summarized as frequencies, as well as mean (standard deviation), or median (range). Ethical approval was obtained for the study from the UI/UCH Ethics Committee with approval number UI/EC/22/0460.

## Results

A total of 167 patients with histological diagnoses of breast cancer were recruited over the study period. Patients were aged 19 to 75 years (mean age 46 years), and 128 (76.6%) were premenopausal. All the patients had invasive ductal carcinoma.

Overall, 123 patients underwent a simple mastectomy and axillary clearance surgery while the remaining 44 patients had other surgical interventions (chest tube insertion for patients who had malignant pleural effusion, metastasectomy, tumor biopsy, etc.) being stage IIIC and stage IV breast cancer patients. All patients received chemotherapy comprising neoadjuvant (37.1%), adjuvant (53.8%), and palliative (8.9%) treatments. Tables 1-2 show the age distribution of the patients, menopausal status, and AJCC, respectively, while Tables 3-4 show the intervention and the Caprini risk of the patients, respectively.

**Table 1:** Patient characteristics.

Characteristics	Number of patients, *n*	Percentage (%)
Age (years)		
20-29	2	1.1
30-39	34	20.4
40-49	41	24.6
50-59	68	40.7
60 and older	22	13.2
Menopausal status		
Premenopause	128	76.6
Menopause	39	23.4

**Table 2:** AJCC classification of patients. AJCC, American Joint Committee on Cancer

AJCC disease stage	Number of patients			Percentage (%)
I		-	-	
IIA		5	3	
IIB		27	16	
IIIA		28	16.8	
IIIB		63	37.8	
IIIC		12	7.2	
IV		32	19.2	

**Table 3:** Treatment given.

Treatment	Number of patients, *n*	Percentage (%)
Surgical treatment		
Simple mastectomy and axillary clearance	123	74
No surgery	44	26
Chemotherapy		
Neoadjuvant	62	37.1
Adjuvant	90	53.8
Palliative	15	8.9

**Table 4:** Caprini risk category.

Caprini risk category	Number of patients, *n*	Percentage (%)
Very low	0	0
Low risk	109	65
Moderate	53	32
High risk	5	3

Caprini risk assessment scores and VTE risk factors

The majority (79%) of premenopausal women were in the low-risk category. Only two patients in this group were at high risk for VTE events. The majority (72%) of postmenopausal women were in the moderate risk category. Heart failure, stroke, family history of DVT, and hypertension were uncommon. Only 51 patients (30.5%) were overweight. There was no history of previous DVT in any of the patients. Table 4 summarizes the Caprini risk category.

VTE prophylaxis and outcomes

There were no reported side effects from the use of chemical thromboprophylaxis during the study.

VTE prophylaxis did not prevent fatal VTE in two patients who underwent a simple mastectomy. The first patient (AJCC IIIA) was a low risk by assessment but had a sudden pulmonary embolism (PE) on the third postoperative day, and the autopsy confirmed a saddle embolism. The second patient (AJCC IIIB) was at moderate risk by assessment and developed symptoms of a DVT four days postoperatively, which was confirmed with a doppler Ultrasound scan. The hematologist modified the dosage from a prophylactic to a therapeutic dose. However, the patient experienced a progressive deterioration of the cardiorespiratory system, leading to death on the 13th day following diagnosis.

## Discussion

Little is known about the burden of VTE in patients who are treated on clinical pathways in low-economic settings. To our knowledge, this study is one of the first series in Nigeria to evaluate VTE prophylaxis and treatment in breast cancer patients undergoing surgery and will aid in establishing recommendations for its routine implementation.

The rate of VTE in this study is 1.2%, which is comparable to the global incidence of 0.8% to 10% [4,5,7,8,9]. The factors responsible for this seem to differ between developing countries like ours and developed countries. Women with breast cancer in developed countries are mostly postmenopausal and elderly with comorbidities [5,10-12]; however, improved breast cancer awareness and access to screening avoids presentation at an advanced stage and ensures less extensive surgery and subsequently a low rate of VTE [4]. Our patients are predominantly premenopausal younger women with few comorbidities and, therefore, have reduced intrinsic risk factors for VTE.

Limited access to specialized diagnostic tests, including CT angiography and ventilation-perfusion (VQ) scanning, may partly explain the low rate of VTE diagnosis in our setting. However, both VTE events in this study resulted in mortality. This is consistent with the reported higher VTE-associated mortality in black compared to white women [11,13].

Patient tumor and cancer treatment characteristics also influence the occurrence of VTE events. While presentation with relatively more advanced disease is common in our cohort, the association between tumor biology and VTE is a late-onset event. The impact of surgery and systemic chemotherapy is limited to early-onset events. Globally, VTE risks associated with general surgical procedures have been consistently downgraded with an overall reduced risk and reporting of VTE episodes [14]. Still, risk stratification is useful for determining appropriate thromboprophylaxis for VTE [15]. The Caprini score risk assessment model has been validated for patients undergoing surgery [15,16]. Its implementation should inform a decision on the use of systemic thromboprophylaxis for hospitalized patients at high risk for VTE [4,17].

Simple mastectomy with axillary clearance is the most common surgical procedure for breast cancer in our cohort as the majority of patients present with advanced-stage disease, and this surgery may exacerbate VTE risk [14]. Central venous catheters (CVC), interventional radiology, and angioembolization treatment procedures are all known to contribute to the increased risk of VTE in breast cancer but are not routinely available in our low-resource setting.

Obesity, a well-known patient-related risk factor for VTE with a moderate association [18], was a frequent characteristic among patients in this study. The pathophysiology of VTE in obese individuals has been associated with decreased venous return [19,20], endothelial dysfunction, and elevated levels of coagulation factors [21,22]. Obesity, in association with other risk factors, poses greater risks for developing VTE [18,23]. Although the majority of the patients were in the low-risk category, nearly a third of them were overweight. Weight impacts the administration of LMWH because LMWHs are not distributed in fat [24,25]. This situation can potentially result in underdosing, leading to a loss of efficacy, or excessive administration of LMWH in overweight patients, increasing the risk of bleeding complications [25]. Thus, concerns for optimal dosing of LMWH in patients with obesity and breast cancer may have significant implications for clinical practice. The absence of side effects or complications in our study suggests a good safety profile [26,27]. A unique feature of VTE treatment in patients with cancer is the preferred use of LMWH over other forms of anticoagulant therapy. Although there is no consensus on the appropriate duration of LMWH use, it is associated with low mortality after a VTE event [9].

It has been reported that blacks have a 30% to 60% higher risk of developing VTE compared to whites [11]. Further epigenetic studies to understand the reasons for low VTE risk and rising breast cancer prevalence are indicated as there is a dearth of knowledge on the genetic predisposition to VTE among Africans.

Recent surgery is a known significant risk factor for VTE [12,28,29], and majority of our patients had surgery. Despite the absence of sophisticated VTE prevention and diagnostic tools, such as pneumatic compression devices, d-Dimer assay, impedance plethysmography, and ventilation-perfusion scan in our setting, the incidence of VTE is within acceptable limits. However, understanding the risk of VTE in breast cancer is important for institutionalizing appropriate VTE prevention protocols.

Our study provides evidence for individualized VTE prophylaxis and may obviate the need for pharmacologic prophylaxis in low-risk patients.

The small sample size in this study limits a more robust conclusion. 

Many of the patients underwent mastectomy without breast reconstruction, which is a limitation of this study. This accounts for shorter operating times in this study, which may impact rates of VTE. In a low-income country like ours, few patients can afford plastic surgery while facilities like pneumatic pumps and venous filters are lacking. The rates of VTE among more affluent patients may match findings in developed countries.

## Conclusions

VTE is a rare event in hospitalized patients with surgical breast cancer receiving thromboprophylaxis. The implementation of routine VTE prophylaxis is safe and feasible but adds to the burden of breast cancer care in resource-poor settings. Patient-related factors appear to be less contributory to VTE risk in our study. The assessment of risk factors via the use of the risk assessment tool (Caprini) will help identify patients with very low risk who may not require pharmacologic VTE prophylaxis protocols.
